# Immunothrombosis in Sepsis: Cellular Crosstalk, Molecular Triggers, and Therapeutic Opportunities—A Review

**DOI:** 10.3390/ijms26136114

**Published:** 2025-06-25

**Authors:** Addis Aklilu, Michael Siu-Lun Lai, Zhiwei Jiang, Shea Ping Yip, Chien-Ling Huang

**Affiliations:** Department of Health Technology and Informatics, The Hong Kong Polytechnic University, Hong Kong, China; addis.belete@connect.polyu.hk (A.A.); lun.lai@cevr.hk (M.S.-L.L.); zhiwei.jiang@connect.polyu.hk (Z.J.)

**Keywords:** immunothrombosis, sepsis, cellular crosstalk, signaling cascades, therapeutic strategies, NETosis, disseminated intravascular coagulation (DIC)

## Abstract

Sepsis remains a critical global health challenge characterized by life-threatening organ dysfunction arising from a dysregulated host response to infection. Immunothrombosis refers to the intersection of immune activation and coagulation pathways, particularly relevant in the context of sepsis. A growing body of evidence identifies immunothrombosis, a tightly interwoven process between innate immunity and coagulation. While immunothrombosis serves as a host defense mechanism under physiological conditions, its aberrant activation in sepsis precipitates microvascular thrombosis, organ ischemia, and progression toward disseminated intravascular coagulation (DIC). This review provides a comprehensive overview of the cellular contributors to immunothrombosis, including neutrophils, monocytes, platelets, and endothelial cells, and elucidates the signaling cascades, such as nuclear factor kappa B (NF-κB), mitogen-activated protein kinase (MAPK), and inflammasome activation, that govern their interplay. We further highlight emerging molecular mediators, including extracellular traps, tissue factor expression, and cytokine amplification loops, that collectively promote pathological thromboinflammation. A deeper understanding of these interconnected pathways offers critical insights into the pathogenesis of sepsis and unveils potential targets for timely intervention. Ultimately, this review aims to bridge immunological and hematological perspectives to inform the development of novel therapeutic strategies against sepsis-induced coagulopathy.

## 1. Introduction

Sepsis is a serious medical emergency characterized by multiple organ failure resulting from dysregulated inflammatory responses to infectious diseases, posing a significant global health concern [1]. It remains a leading cause of mortality in intensive care units with 250,000 to 300,000 deaths reported annually in the United States alone [2,3]. Although the exact global burden is difficult to ascertain, it has been estimated that over 30 million people are affected by sepsis worldwide each year with approximately six million deaths annually [4]. A more recent report recorded even higher figures with 48.9 million incident cases and 11 million deaths globally [5].

Sepsis is classified as a systemic inflammatory response syndrome (SIRS) triggered by infections [6] with pathogenic bacterial, parasitic, or viral agents [7]. Both bacterial infections and viral pathogens, such as the coronavirus that is responsible for coronavirus disease 2019 (COVID-19), can induce sepsis, often leading to severe clinical conditions that necessitate immediate hospitalization [8]. Sepsis is marked by two types of immune dysregulation: hyperinflammation (commonly referred to as a cytokine storm) and immunosuppression. The excessive inflammatory response can activate the coagulation cascade, and this hyperactivation may result in the formation of intravascular microthrombi, further complicating the infection [9].

During the early phase of infection, coagulation plays a protective role by containing pathogens locally through the formation of fibrin clots, thereby limiting the dissemination of the invading pathogens into systemic circulation [10]. However, in severe sepsis, overwhelming cytokine production at the site of infection triggers the coagulation system, promoting procoagulant molecule synthesis and suppressing anticoagulant mechanisms, ultimately leading to widespread formation of micro- and macrothrombi [6].

**Figure 1:**
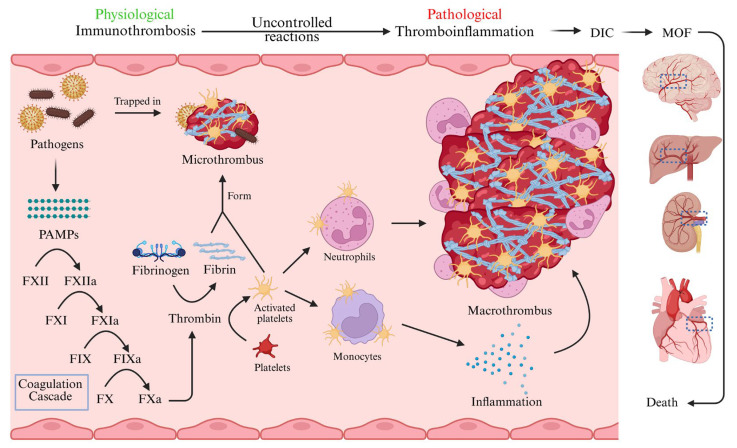
Immunothrombosis and thromboinflammation in septic infection. Immunothrombosis is a physiological protective mechanism that traps pathogens and prevents their spread in circulation. The PAMPs expressed by pathogens will trigger the activation of coagulation factor XII (FXII). The activated FXII (FXIIa) further initiates the coagulation cascade, eventually leading to the formation of thrombin. Thrombin will then activate platelets and cleave fibrinogen into fibrin. Fibrin and activated platelets form the microthrombus to trap the pathogens. However, thromboinflammation occurs when immunothrombosis is uncontrolled. In this pathological condition, the excessively activated platelets will interact with monocytes and neutrophils. These interactions induce the secretion of proinflammatory cytokines and the formation of macrothrombi. These large blood clots may cause disseminated intravascular coagulation (DIC), eventually blocking the blood vessels of different organs and resulting in multi-organ failure (MOF). If the affected organs are vital to survival, such as the brain, liver, kidney, and heart, it will eventually cause death. PAMPs, pathogen-associated molecular patterns; FXII, coagulation factor XII; FXIIa, activated coagulation factor XII; FXI, coagulation factor XI; FXIa, activated coagulation factor XI; FIX, coagulation factor IX; FIXa, activated coagulation factor IX; FX, coagulation factor X; FXa, activated coagulation factor X; DIC, disseminated intravascular coagulation; MOF, multi-organ failure. (Created in BioRender. Huang, C. (2025) https://BioRender.com/3370ji3 accessed on 17 June 2025).

Coagulation abnormalities are commonly observed in nearly all individuals with sepsis [1]. Dysregulated activation of the coagulation system can result in sustained thrombus formation within the vasculature, a phenomenon referred to as immunothrombosis. This process involves the physiological generation of microthrombi through coordinated interactions among innate immune cells, platelets, and coagulation factors, serving as an early defense mechanism to contain pathogens and prevent their systemic spread (Figure 1). This process is often described as a double-edged sword due to its dual protective and pathological roles [11]. While immunothrombosis restricts pathogen spread and offers localized defense, it also results in tissue damage through compromised microcirculation and ischemia [12]. The process is initiated by components of innate immunity, which constitute the first line of defense and play a pivotal role in the early resolution of infection. Immunothrombotic clots consist of interwoven fibrin matrices formed by the concerted action of coagulation factors, platelets, and leukocytes [13]. Pathology arises when there is dysregulated or excessive formation of such clots, leading to uncontrolled development of both micro- and macrothrombi, particularly in small blood vessels of vital organs, thus obstructing normal blood flow [11].

This state of dysregulated immunothrombosis, often referred to as thromboinflammation, underlies many of the fatal complications observed in sepsis and other severe coagulation disorders such as disseminated intravascular coagulation (DIC) [10]. The pathophysiology of sepsis-associated coagulopathy involves a complex interplay among immune system dysregulation, heightened inflammatory responses, activation of platelets and coagulation pathways, endothelial injury, and imbalance in the coagulation–fibrinolysis axis [1]. In this review, we explore the cellular activation events involved in initiating and propagating microvascular clot formation, the intercellular interactions and signaling pathways, and the molecular mechanisms that drive thrombosis in the context of sepsis. We also discuss current and emerging therapeutic strategies for mitigating sepsis-induced thromboinflammation.

## 2. Pathophysiology of Sepsis-Induced Immunothrombosis

Immunothrombosis is an essential component of the host’s innate defense mechanism against various exogenous and endogenous stimuli, yet it results in the formation of large and small thrombi in pathological conditions such as acute injury and critical illness, including sepsis [14]. The immune response to sepsis involves a complex interplay between immune activation and the coagulation cascade. While inflammation and coagulation are fundamentally protective in controlling microbial proliferation and facilitating pathogen clearance, an excessive inflammatory response and sustained activation of coagulation can lead to tissue injury and multiple organ dysfunction [15].

### 2.1. Sepsis-Induced Hyperinflammation

Prolonged and excessive immune responses, particularly when unregulated, can result in tissue damage and disruption of vascular homeostasis. Upon pathogen recognition, innate immune cells detect pathogen-associated molecular patterns (PAMPs) via pattern recognition receptors, such as toll-like receptors (TLRs), triggering cytokine and chemokine release, and complement system activation [16]. The proinflammatory cytokines produced during this cascade contribute to widespread inflammation and cellular dysfunction [17]. During infection, the immune and coagulation systems return to a normal state through the homeostatic system. Unless the pathogens are removed from the body and tissue damage is ceased, the persistent inflammatory state in sepsis stems from continuous exposure to both damage-associated molecular patterns (DAMPs) and PAMPs, leading to the recruitment and activation of leukocytes (such as neutrophils), endothelial activation, and complement involvement. During the early phase of sepsis, acute-phase reactants like C-reactive protein and inflammatory cytokines surge, amplifying the immune response. The massive release of proinflammatory cytokines, such as tumor necrosis factor (TNF) and interleukin-1β (IL-1β), can result in a cytokine storm in the bloodstream (hypercytokinemia) [18], which substantially contributes to the activation of the coagulation cascade by enhancing procoagulant production and downregulating anticoagulant pathways [15,19].

### 2.2. Sepsis-Induced Immunothrombosis

The excessive inflammatory response in sepsis leads to tissue injury and disrupts the coagulation–fibrinolysis balance, resulting in uncontrolled clot formation within the vasculature, commonly referred to as DIC [20]. The coordinated activation of coagulation factors, platelets, and immune cells (e.g., neutrophils and lymphocytes) along with endothelial damage plays a central role in sepsis-induced coagulopathy and DIC [9]. Fibrinogen, a crucial molecule in coagulation and inflammation, is elevated in sepsis and represents the intricate connection between physiological and pathological processes. During coagulation, fibrinogen is converted into fibrin by thrombin, forming a structural mesh that stabilizes blood clots and entraps pathogens. Although this mechanism is protective, excessive fibrinogen levels (hyperfibrinogenemia) and aberrant clot formation contribute to the hypercoagulable state in sepsis [21]. Elevated fibrinogen levels in tissues or circulation, along with other coagulation factors, can impair the fibrinolytic system. While balanced fibrinolysis supports pathogen containment through clot formation, sepsis often disrupts this equilibrium, leading to excessive coagulation and reduced clot resolution [22]. Consequently, sepsis-induced coagulopathy (SIC) is marked by heightened coagulation factor activity, endothelial dysfunction, and disruption of the coagulation–fibrinolysis equilibrium, leading to excessive thrombus formation [23].

### 2.3. Crosstalk Between Inflammation and Coagulation

The interplay between inflammation and coagulation is bidirectional: inflammation triggers coagulation, and coagulation further amplifies inflammatory responses [24]. Immune cells, along with proinflammatory cytokines and chemokines, initiate procoagulant activity while simultaneously inhibiting anticoagulant pathways. The coagulation cascade is initially activated by tissue factor (TF) expression on activated monocytes and endothelial cells (ECs), and subsequent thrombin generation. Furthermore, the anticoagulant protein C pathway, which mainly occurs via the protein C receptors on ECs, is suppressed by proinflammatory cytokines [25,26]. This interdependent relationship between the immune and the coagulation systems drives the formation of microvascular thrombi during sepsis [19]. TF is widely distributed in blood–tissue barrier compartments, and its exposure is heightened upon endothelial barrier disruption. Inflammatory mediators, such as cytokines and C-reactive protein, induce TF expression during sepsis. This expression is frequently observed on monocytes and macrophages, especially in severe bacterial infections, in the presence of platelets and granulocytes via a P-selectin–dependent pathway [22,27]. In septic conditions, coagulation activation is further exacerbated by compromised anticoagulant activity.

In addition to cytokines and chemokines, other inflammatory molecules, including matrix metalloproteinases, heparanase, hyaluronidase, thrombin, elastase, reactive oxygen species (ROS), and reactive nitrogen species (RNS), damage the endothelial glycocalyx. This disruption alters the expression of key surface receptors and molecules such as TF, adhesion molecules, and von Willebrand factor (VWF). Notably, adhesion molecules, like intercellular adhesion molecule (ICAM), vascular cell adhesion molecule (VCAM), and E-selectin, facilitate the attachment of monocytes, neutrophils, and platelets to the endothelial cell surface, contributing significantly to microthrombus formation [28].

Furthermore, endothelial dysfunction plays a crucial role in the progression of sepsis-induced pathological immunothrombosis. During endothelial damage, glycocalyx destruction facilitates the adhesion and aggregation of immune cells on the endothelium and the activation of coagulation factors [29]. Sepsis is highly associated with procoagulant, proadhesive, and apoptotic activities of endothelial cells. This phenomenon is the essential part of intravascular thrombus formation [30,31].

## 3. Cellular Activation

### 3.1. Cellular Activation in Sepsis-Induced Immunothrombosis

As established through the complex interplay between immune and coagulation systems, innate immune cells and ECs are vital for resolving infections. In this regard, myeloid cells such as monocytes, macrophages, and neutrophils are recognized as key contributors to thromboinflammation via increased TF expression, formation of neutrophil extracellular traps (NETs), signaling pathway activation, and aberrant protease-activated receptor signaling driven by coagulation factors [32]. The involvement of platelets and ECs is also crucial in the development of microthrombosis across several pathological conditions, including severe sepsis [33,34].

### 3.2. Activation of Leukocytes

The activation, differentiation, and proliferation of leukocytes are essential processes in recognizing and eliminating pathogens such as bacteria, viruses, fungi, and parasites [35]. These immune cells are critical components of the host defense system and express a range of receptors, including TLRs, Fcγ-receptors, G-protein-coupled receptors, adhesion receptors, and cytokine receptors, that mediate their response to infections. Among these, TLRs play a central role in detecting microbial and host-derived signals (PAMPs and DAMPs), thereby initiating immune responses [12]. In conditions like sepsis, leukocyte activation involves significant biophysical and biochemical changes that alter their function [36]. Intriguingly, during sepsis, leukocyte numbers may decline, resulting in leukopenia and immunosuppression [37].

#### 3.2.1. Activation of Monocytes

Monocytes and macrophages serve as frontline defenders through phagocytosis and inflammatory mediator production [38]. Activated monocytes express a variety of surface receptors [39] and transition into hyperinflammatory subtypes that secrete cytokines and chemokines to recruit additional immune cells [40,41].

**Figure 2:**
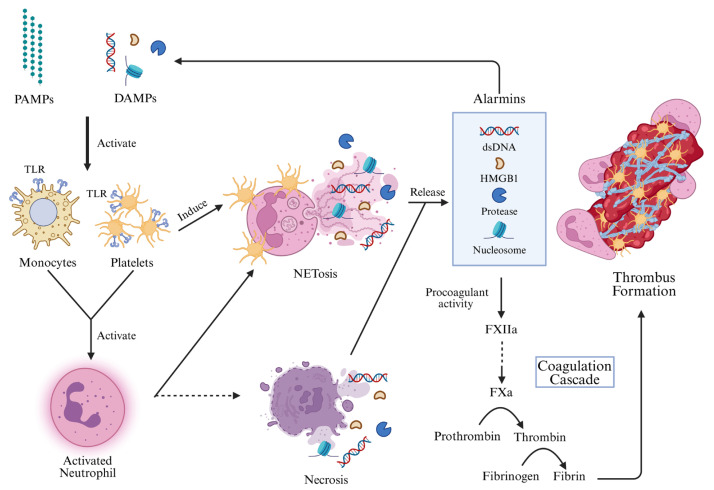
Activation of neutrophils and formation of NETs. When monocytes and platelets detect PAMPs and DAMPs via their TLRs, these cells are activated by the signals. They will then activate the neutrophils. Most of the activated neutrophils will form the neutrophil extracellular traps (NETosis), while a minority of activated neutrophils will undergo necrosis (as represented by the dotted line in the figure). Both NETosis and necrosis release inner cell contents, known as alarmins. Alarmins are molecules released from the cells and hence are one subtype of DAMPs. Therefore, alarmins not only can perform procoagulant activity to activate the coagulation cascade to produce thrombus, but also further activate more monocytes and platelets to amplify the process. PAMPs, pathogen-associated molecular patterns; DAMPs, damage-associated molecular patterns; TLR, Toll-like receptor; NETs, neutrophil extracellular traps; dsDNA, double-stranded DNA; HMGB1, high-mobility group box 1; FXIIa, activated coagulation factor XII; FXa, activated coagulation factor X. (Created in BioRender. Huang, C. (2025) https://BioRender.com/3370ji3 accessed on 17 June 2025).

TLR activation by PAMPs, such as lipopolysaccharide (LPS) and host-derived DAMPs, triggers protease cascades that can result in DIC [42]. When monocytes recognize LPS via TLRs, they release TF, initiating the coagulation cascade and contributing to thrombosis (Figure 2) [38,43]. These cells also release TF-containing extracellular vesicles, which are elevated in endotoxemic mice and septic patients and implicated in inflammasome and coagulation activation [44]. Comparative studies indicate higher TF expression in intermediate and non-classical monocytes among septic patients [43]. Interestingly, non-classical monocytes also display thrombolytic potential and aid neutrophil recruitment during stroke-induced thrombosis [45]. Monocytes in sepsis show elevated expression and release of vascular endothelial growth factor A, indicating enhanced angiogenic activity [38]. Moreover, these cells polarize toward proinflammatory M1 macrophages via NF-κB signaling [46], which promotes cytokine production and cell-to-cell interactions, particularly with platelets and ECs [47,48,49].

#### 3.2.2. Activation of Neutrophils

Neutrophils, or polymorphonuclear cells (PMNs), are abundant first-line innate immune cells. Their recruitment is regulated by chemokine receptors (CXCRs), and their TLR expression is fully upregulated upon maturation. CXCR2 and TLRs indicate mature neutrophils, while CXCR4 is linked to bone marrow retention [50]. TLR2 and TLR4 are especially important for pathogen recognition and chemotaxis [50]. While monocytes/macrophages and platelets were traditionally viewed as primary players in coagulation, neutrophils are now known to play an equally vital role [51].

Neutrophils contribute to NET formation, with NETosis regulated by proteins such as peptidylarginine deiminase I4 (PADI4), which plays a role in both immune responses and coagulation during sepsis-related immunothrombosis [45]. NET-associated coagulation factor activation leads to pathological thromboinflammation [52]. NF-κB drives the production of cytokines such as IL-1 and IL-12, enhancing inflammatory responses. Neutrophils also release NETs composed of DNA fibers and proteins (e.g., neutrophil elastase, myeloperoxidase, and cathepsin G), which trap pathogens and may also activate coagulation factors and promote thrombosis [53].

NETs trigger a proinflammatory and procoagulant endothelial phenotype by inhibiting anticoagulation and inducing TF expression [54]. They also contribute to platelet-driven coagulation, leading to endothelial dysfunction and coagulopathy in sepsis [55], and play a role in venous thrombosis [56]. The presence of TF-enriched NETs in septic patients and animal models further supports their role in pathological immunothrombosis (Figure 2) [57].

### 3.3. Activation of Platelets

Platelets, though anucleate, are key regulators of hemostasis and thrombosis [58]. Emerging evidence suggests that they actively participate in immune modulation and immunothrombosis [59]. Platelets express TLRs, complement receptors, Fc receptors, and NOD-like receptors, enabling them to produce immunomodulatory molecules that influence both innate and adaptive immunity [60,61,62,63].

Infections, including bacterial and viral sepsis, activate platelets, contributing to pathological thrombosis and DIC, ultimately leading to MOF [64]. Platelets are highly responsive to inflammatory and procoagulant signals [65]. Activation induces synthesis of thromboxane A2 and adenosine diphosphate (ADP) from delta-granules, promoting platelet aggregation [66]. Upon activation, α-granules fuse with the platelet membrane to release proteins, and ADP-rich granules further amplify activation via P2Y1 and P2Y12 receptors (receptors for ADP). P-selectin, an inflammatory marker, binds PSGL-1 to mediate adhesion with monocytes, neutrophils, and ECs [65].

**Figure 3:**
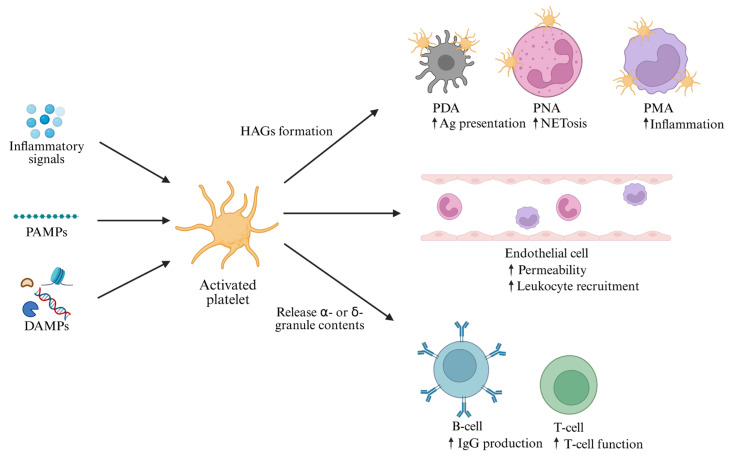
Platelet activation and its interactions with various immune cells and endothelial cells. Different signals, including cytokines, PAMPs and DAMPs, can activate platelets. Firstly, the activated platelets can bind to different cell types other than platelets through adhesion molecules, which is known as heterotypic aggregation. The heterotypic aggregates (HAGs) typically form through the interaction of platelets with dendritic cells, neutrophils, or monocytes, giving rise to PDA, PNA and PMA, respectively. Secondly, activated platelets can interact with endothelial cells to increase endothelium permeability and recruit more leukocytes. Thirdly, activated platelets will release α- or δ-granule contents to enhance the activities of B-cells and T-cells. PAMPs, pathogen-associated molecular patterns; DAMPs, damage-associated molecular patterns; HAGs; heterotypic aggregates; PDA, platelet-dendritic aggregation; PNA, platelet–neutrophil aggregation; PMA, platelet–monocyte aggregation; Ag, antigen. (Created in BioRender. Huang, C. (2025) https://BioRender.com/3370ji3 accessed on 17 June 2025).

Hyperinflammation in sepsis promotes platelet–monocyte aggregation (PMA) [48] and platelet–neutrophil aggregation (PNA) [67,68]. These aggregates are stabilized by P-selectin and other molecules (Figure 3). TLR4-mediated interactions between platelets and neutrophils can lead to NET dysregulation, causing tissue injury and immunothrombosis [60,69]. Additionally, platelet-derived thrombin promotes further coagulation and TF expression, reinforcing the clotting cascade and NET formation [70,71]. In severe sepsis, platelets are rapidly consumed, resulting in thrombocytopenia [58], which may impair monocyte function [72] and increase bleeding risk [73,74].

### 3.4. Activation of ECs

ECs maintain vascular homeostasis by expressing anti-inflammatory and antithrombotic molecules [33]. They are among the first to encounter microbes and inflammatory mediators in sepsis [31]. ECs can be directly activated by microbial PAMPs or indirectly by NETs and cytokines such as TNF-α, IL-6, and IL-1 [33,75].

Similar to immune cells, ECs detect PAMPs through TLRs and respond by expressing adhesion molecules like ICAM-1, VCAM-1, E-selectin, P-selectin, and VWF, which are associated with disease severity and mortality [29]. Activation leads to the exposure of VWF and integrins, facilitating thrombus formation with platelets and coagulation factors [76]. NF-κB is a central regulator of cytokine expression and EC activation in sepsis [31,77,78].

The endothelial glycocalyx, primarily composed of heparan sulfate, is a vital structure that maintains vascular surface integrity and regulates cellular trafficking [77]. In infectious states, particularly sepsis, damage to the glycocalyx, combined with EC injury and the breakdown of intercellular junctions, result in increased vascular permeability and interstitial fluid accumulation [79]. This disruption not only contributes to tissue edema but also facilitates the adhesion of leukocytes and platelets, creating favorable conditions for the formation of cellular aggregates and intravascular thrombosis [29]. Persistent inflammation and oxidative stress are major contributors to glycocalyx degradation during septic progression [80].

**Figure 4:**
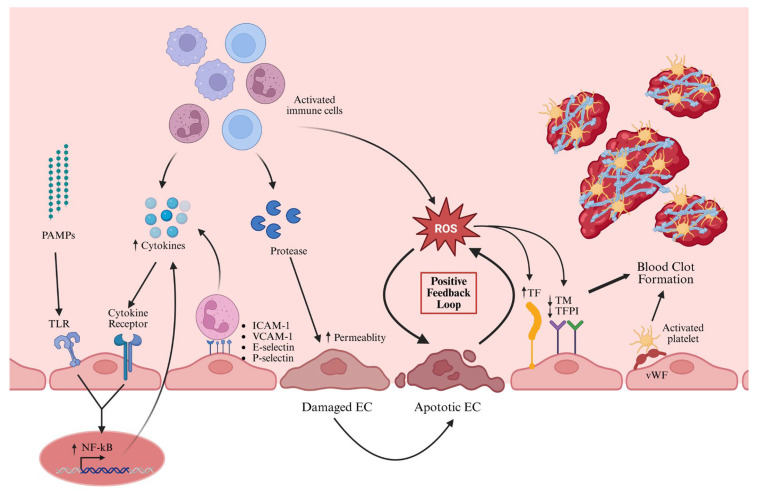
Activation of endothelial cells with their respective interactions with immune cells, molecules and factors during sepsis. ECs can be activated by PAMPs and cytokines through TLR and cytokine receptors, respectively. This activates the transcription factor NF-kB for higher expression of proinflammatory cytokines. The activated ECs also express von Willebrand factor for platelet binding, together with ICAM-1, VCAM-1, E-selectin and P-selectin for leukocyte recruitment and binding. The recruited immune cells will secrete proteases and ROS to increase endothelial permeability and induce EC apoptosis. The apoptotic ECs will also secrete ROS, which can elevate procoagulant TF expression and diminish anticoagulant TM and TFPI expressions. Together with the activated platelets, the thrombus formation is facilitated. PAMPs, pathogen-associated molecular patterns; TLR, Toll-like receptor; NF-kB, nuclear factor kappa B; ICAM-1, intercellular adhesion molecule 1; VCAM-1, vascular cell adhesion molecule 1; EC, endothelial cell; ROS, reactive oxidative species; TF, tissue factor; TM, thrombomodulin; TFPI, tissue factor pathway inhibitor; vWF, von Willebrand factor. (Created in BioRender. Huang, C. (2025) https://BioRender.com/3370ji3 accessed on 17 June 2025).

Glycocalyx degradation and increased ICAM/VCAM expression enhance leukocyte recruitment to injury sites [31]. ECs lose their anticoagulant functions and release VWF from Weibel–Palade bodies, promoting platelet adhesion. In sepsis, ECs form ultra-large-VWF-mediated projections that recruit platelets and generate microthrombus strings (Figure 4) [81]. Inflammatory cytokines and immune aggregates upregulate TF expression, amplifying coagulation [30].

ROS, RNS, and proapoptotic mediators further damage ECs in sepsis. Apoptotic ECs display elevated TF, reduced thrombomodulin, and heparan sulfate [31], and tissue factor pathway inhibitor (TFPI), exacerbating thrombin generation and coagulation [79]. EC-derived chemokines and receptors mediate the recruitment of leukocytes and platelets, driving thromboinflammation and immunothrombosis [82]. The cumulative impact of EC activation culminates in vascular dysfunction and MOF [29,80].

## 4. Cellular Signaling Pathways in Immunothrombosis

In the presence of complex cellular interactions and signal transduction among immune cells and ECs, an unbalanced immune response contributes to the multifaceted and fatal nature of sepsis. Sepsis is initiated by the detection of PAMPs or DAMPs via TLRs present on the surface of infected cells, triggering the activation of cellular signaling pathways [83]. SIRS, a hallmark of severe sepsis, arises from hyperinflammatory responses to infectious agents and results in multiple organ dysfunction/failure and disrupted homeostasis. This is mediated through inflammatory and coagulation signaling pathways, including NOD-like receptor protein 3 (NLRP3) inflammasomes, Janus kinase-signal transducer and activator of transcription (JAK-STAT), NF-κB, MAPK, and cyclic GMP-AMP synthase-stimulator of interferon genes (cGAS-STING) among others. Various cell types utilize different signaling pathways and molecular mediators to interact with surrounding cells and to propagate intracellular signaling events (Table 1) [47].

### 4.1. NLRP3 Inflammasome Pathway

One of the pivotal signaling mechanisms is the NLRP3 inflammasome pathway, which plays an essential role in venous thrombosis. It responds rapidly to PAMPs and DAMPs, eliciting a proinflammatory cascade through caspase-1 activation and the subsequent release of IL-1β and IL-18 [84,85,86]. Canonical and non-canonical activation of the NLRP3 inflammasome through caspase-1 and caspase-11, respectively, also promotes TF release via pyroptotic pores, serving as a central driver of immunothrombosis [10,87]. Once released, TF initiates the coagulation cascade by activating FVII to FVIIa, which subsequently activates FX to FXa, propagating via the common pathway and thrombin generation required for thrombus formation [10,88].

Recent studies have revealed that NLRP3 inflammasome activation can be further amplified by other pathways, including the cGAS-STING axis [89] and NF-κB signaling [46]. The NF-κB pathway can be stimulated by a broad array of signals, including microbial components (e.g., PAMPs), cytokines, and physical stress such as radiation, growth factors, ROS, and carcinogenic stimuli. It plays a crucial role in inflammatory signaling and links inflammation to coagulation activation, thereby contributing to thrombus development [90].

### 4.2. cGAS-STING Pathway

In addition, the cGAS-STING pathway is activated by intracellular DNA abnormalities and plays a vital role in inflammatory responses [91,92]. In sepsis, STING activation triggers both inflammatory signaling and coagulation dysregulation. Activation of gasdermin D, an effector protein that mediates pyroptosis via pore formation on cellular membranes, promotes the release of TF, which initiates the coagulation cascade [91]. Moreover, the STING–IRF3–NF-κB axis becomes active during sepsis, alongside marked increases in circulating cell-free DNA. Mitochondrial DNA (mtDNA), in particular, is more strongly associated with disease severity and mortality than nuclear DNA [83]. These cell-free DNA molecules intensify inflammatory responses through STING interaction. Furthermore, interferon regulatory factor 3 (IRF3), NF-κB, and STING form complexes with TANK-binding kinase 1, thereby enhancing transcriptional activity through phosphorylation of additional transcription factors [93].

### 4.3. Other Signaling Pathways

Another key pathway involved in platelet activation and thrombosis is the phosphatidylinositol 3-kinase/protein kinase B (PI3K/Akt) signaling axis, particularly in integrin inside-out signaling. Platelet integrins contribute significantly to hemostasis and thrombus formation by mediating intra- and intercellular interactions. The PI3K/Akt pathway regulates both inside-out and outside-in signaling events necessary for platelet aggregation and stabilization [94,95]. Moreover, the PI3K/Akt complex, in conjunction with HIF-1α on PMNs, regulates glycolysis under septic conditions [96].

Neutrophils also contribute significantly to thrombosis via the production of NETs. NET release is stimulated by various agents, including LPS, IL-8, phorbol 12-myristate 13-acetate, interferons, activated platelets, ECs, and plasma-derived proteins from septic patients [97,98]. The constituents of NETs, including histones, DNA, and elastase, enhance immunothrombosis by interacting with coagulation machinery. Histones activate platelets and thrombin generation; DNA activates factor XII; and elastase degrades coagulation inhibitors, thereby supporting clot formation. NETs play a fundamental role in thrombosis by interacting with ECs, platelets, red blood cells, and coagulation factors [97,99].

Overall, innate immune cells, particularly monocytes/macrophages, neutrophils, and platelets, are critical for inflammasome activation and proinflammatory cytokine production. During the early stages of thrombus formation, IL-1α released from injured vascular ECs facilitates the recruitment and activation of macrophages, neutrophils, and platelets at the site of thrombus development [88].

**Table 1:** Key signaling molecules involved in cellular interaction and pathways during sepsis-induced immunothrombosis.

Type of Molecules	Source of Cells	Pathways Involved in	Roles in the Thrombosis Formation	References
Phosphatidylinositol 3-kinases (PI3Ks)	Platelets	PI3K/Akt pathway	Phosphorylates phosphoinositide lipids at the 3 position of the inositol ring and regulates cell growth.	[94]
Integrin α_IIb_β_3_	Platelets	PI3K/Akt pathway	Initiates intracellular signaling pathways for platelet activation and is involved in establishing cell–cell contact during platelet aggregation and thrombus formation.	[94]
FcγRIIA	Platelets (abundant), monocytes, neutrophils	In different pathways	Major transmembrane signaling adapter for α_IIb_β_3_ outside-in signaling pathway in thrombosis.	[100]
cGAS	All cells	cGAS-STING pathway	cGAS detects aberrant abnormal DNAs, and activated cGAS initiates the synthesis of a signaling molecule known as cGAMP in which formation of 2′-3′-cGAMP from ATP and GTP. In turn it binds STING.	[91,92]
STING	All cells	cGAS-STING pathway	Activated STING prompts the production of interferons and proinflammatory factors, including TNF-α, IL-6, among others, through the activation of transcription factors like IRF3 and NF-κB.	[91,92]
NLRP3	Macrophages/monocytes/Tohoko Hospital pediatrics 1 (THP1)	Inflammasome pathway, cGAS-STING pathway	Promotes the production of proinflammatory cytokines through activation of caspases.	[86,88]
Cytosolic DNA	Damaged mitochondria	cGAS-STING pathway, inflammasome pathway	Activates NLRP3 through upregulating the cGAS-STING axis.	[89]
NF-kB	All cells	NF-kB signaling pathway and other pathways	A crucial transcription factor involved in the expression of genes for the activation of the inflammatory response and homeostasis.	[46,101]
TF	Monocytes, ECs	Extrinsic coagulation pathway	Complexes with FVII/VIIa to proteolytically activate factors IX to IXa and X to Xa, resulting in thrombin generation.	[10]
Thrombin	Enzymatic cleavage of prothrombin	Common pathway of coagulation	Activates protease-activated receptors, which are critical for the interplay between inflammation and coagulation, boosting proinflammatory cytokine secretion and activating platelets. Converts fibrinogen to fibrin for clot formation. Involved in NETosis together with activated platelets and neutrophils.	[10]
NETs	Neutrophils	PAD4 pathway	Trigger a proinflammatory and procoagulant endothelial phenotype by inhibiting anticoagulation and inducing TF expression. Platelet-mediated coagulation activation.	[52,54]

## 5. Molecular Mechanisms of Sepsis-Induced Immunothrombosis

Immunothrombosis represents the intersection of inflammatory and coagulation pathways and is often described as a double-edged sword. While it plays a vital role in containing invading pathogens, dysregulated immunothrombosis is closely associated with severe complications, pathological thrombus formation, and increased mortality [1,102]. Platelets, leukocytes, and ECs are pivotal to this process [103]. The formation of immunothrombosis under abnormal conditions is driven by inflammatory imbalances, platelet and coagulation cascade activation, TF expression, endothelial injury, NETosis, and various immunological and coagulative disruptions [1].

Among the molecular regulators of immune responses and systemic homeostasis, non-coding RNAs, especially microRNAs (miRNAs), are prominent. These small RNAs regulate intracellular signaling cascades, including those that are downstream of TLRs, to prevent excessive inflammatory responses during infection. Dysregulated miRNA expression has been implicated in the severity and progression of sepsis [104]. For example, miR-146a is a key regulator of TLR4 expression in myeloid cells (mainly PMNs and monocytes/macrophages) and plays a role in NET formation and immunothrombosis [102]. Additional miRNAs, such as miR-15a, miR-16, miR-122, miR-150, and miR-223, have been associated with disease severity and poor prognosis in sepsis [104]. Furthermore, hsa-miR-451a has been identified as a major contributor to venous thrombosis in clinical patients [105]. Moreover, the dysregulation of miRNAs in COVID-19 sepsis is associated with increased thromboinflammation [106,107].

Other miRNAs, such as miR-223 and miR-24, along with long non-coding RNAs (lncRNAs), modulate the PI3K/AKT pathway and influence thrombosis and other metabolic functions [108]. LncRNAs have also been shown to drive sepsis pathogenesis by inducing pyroptosis—an inflammatory form of programmed cell death—and inflammasome activation, while promoting immunothrombosis through TF exposure on endothelial cell surfaces [109]. Additionally, various circular RNAs and miRNAs have been investigated for their roles in sepsis-related complications, including multi-organ injury. Emerging studies have highlighted the clinical relevance of these small RNAs in modulating inflammatory and thrombotic pathways [109,110,111].

Exosomal and miRNA-mediated regulations of macrophage polarization have also been noted in septic patients. These regulatory interactions facilitate intercellular communication and contribute to disease progression [112]. Dysregulation of both intracellular and extracellular miRNAs has been reported in clinical and experimental models of sepsis. These molecules influence multiple signaling cascades, including vascular endothelial growth factor, LPS-stimulated MAPK, and NF-κB pathways [113]. Exosome-derived miRNAs specifically modulate inflammation by targeting immune cells and transcriptional regulators such as STAT and NF-κB. In doing so, they contribute to coagulation cascade activation and immunothrombotic progression, underscoring their functional significance [114].

Aberrant miRNA expression in critically ill patients has been linked to immunothrombosis and thromboinflammation via regulation of IL-8, ROS production, and enzymes associated with NETosis [106]. Additionally, miRNAs are involved in endothelial dysfunction, platelet activation, disrupted fibrinolysis, and elevated levels of procoagulant factors [115]. Specific miRNAs regulate various homeostatic processes, such as TF expression (miR-19b/c, miR-126, and miR-145), coagulation factor XI (miR-181a-5p), fibrinogen synthesis (miR-29a/b/c), and anticoagulant proteins like protein S (miR-494) [116]. Inflammatory miRNAs are also considered potential contributors to cardiovascular diseases, including thromboinflammation [117].

Another crucial mechanism in sepsis-associated immunothrombosis is the release of extracellular vesicles from immune cells such as monocytes, macrophages, platelets, and ECs. These vesicles are implicated in the activation of proinflammatory cytokines, procoagulant factors, and thrombosis during sepsis and other infections like COVID-19 [118]. Extracellular histones, when released into circulation, can also stimulate TF expression on ECs via TLR, NF-κB, and AP-1 signaling pathways [119]. Moreover, cell-free mtDNA is released under conditions such as infection, mechanical stress, or environmental injury and is recognized as a potent DAMP in sepsis. Compared to nuclear DNA, mtDNA more strongly activates immune responses via TLR signaling pathways. It plays a significant role in driving immune cell activation and recruitment, shaping the inflammatory response trajectory, and complicating recovery in various forms of sepsis [120].

More importantly, multiple miRNAs identified in both patient samples and experimental sepsis models are involved not only in the pathogenesis of sepsis and septic shock but also in promoting hypercoagulation by upregulating cytokines, chemokines, and procoagulant pathways [121].

Identifying specific molecular drivers and reliable biomarkers is essential for the early detection of sepsis-associated immunothrombosis and related coagulation abnormalities [122,123,124]. Multiple studies have uncovered promising diagnostic biomarkers for accurately identifying patients with SIC. For example, a recent investigation reported approximately twenty coagulation-related genes expressed in sepsis and utilized their expression patterns to construct a deep learning-based predictive model [123]. Moreover, abnormalities in platelet subpopulations identified through transcriptomic profiling have revealed key biomarkers that may support early sepsis diagnosis and assist in monitoring patient prognosis. Platelets play a central role in the development of coagulopathy linked to sepsis and other inflammatory conditions. Thus, gaining deeper insight into their cellular and molecular behavior is essential for improving patient management and clinical outcomes [125]. In addition, platelet-related coagulation disturbances are now recognized as key components in clinical diagnosis, owing to the crucial role of platelets in both immune responses and coagulation processes. Recent advances in bioinformatics have provided valuable datasets that enhance diagnostic precision and support more informed therapeutic decision-making [124,125]. A recent meta-analysis reported that, despite observed heterogeneity across studies, miRNA-223a exhibits high diagnostic accuracy and holds clinical promise for sepsis identification in routine practice [126,127].

In addition, prognostic genomic profiles have been identified to play an important role in patient prognosis [128]. Moreover, some biomarkers in COVID-19 viral sepsis patients are important predictors of thrombosis [129,130,131,132].

Furthermore, unravelling of cell-specific or systemic regulation of immune response and coagulation cascades can help us in the development of appropriate therapeutic strategies. Targeting molecular triggers or biomarkers in different pathways of this disease is an essential alternative in the treatment of septic patients at different stages [133,134]. The therapeutic alternatives of immunothrombosis induced by sepsis are presented in details.

## 6. Therapeutic Strategies for Sepsis-Induced Immunothrombosis

Since the pathophysiologic nature of immunothrombosis is diverse and multifactorial, its treatment remains challenging. There is currently no single effective and safe treatment modality for this condition. Several studies have been conducted to explore therapeutic agents using different strategies. In consideration of the central role of coagulation in promoting microvascular thrombosis and inflammation, the use of therapies that inhibit coagulation may help improve microvascular perfusion, reduce inflammation, and preserve organ function. Most of the antithrombotic treatments currently in practice are anticoagulants that target procoagulant and proinflammatory molecules (Table 2 and Table 3). Septic patients with immunothrombotic complications are conventionally treated with a combination of antibiotics and anticoagulants. However, the prolonged administration of anticoagulant agents may lead to bleeding complications [10].

The most extensively investigated anticoagulant and anti-inflammatory agent in treating coagulation dysfunction is heparin, in both its natural and synthetic forms. Heparin is a part of routine clinical practice and is indicated in various settings, particularly for the prophylaxis and treatment of sepsis-associated coagulopathy and venous thromboembolism [135,136]. Natural anticoagulants, such as activated protein C, antithrombin, TFPI, thrombomodulin, and heparinoids, have also been employed in anticoagulant therapy. A recent study has shown that heparin can also act as an adsorbent molecule for coagulation-related components, including platelet extracellular vesicles, platelet factors, histones, and high-mobility group box 1 (HMGB1) [137]. However, as mentioned earlier, the principal drawback of these treatments in septic patients with thromboinflammation is the elevated risk of bleeding [28,29,138].

Platelet P2Y12 inhibitors, such as clopidogrel and ticagrelor, have been shown to reduce the proinflammatory and prothrombotic effects of P2Y12 in LPS-induced sepsis models [139]. Similarly, glycoprotein IIb/IIIa (GP IIb/IIIa) inhibitors, including prostacyclin, eptifibatide, tirofiban, and aspirin, have demonstrated efficacy in the management of sepsis-associated coagulation complications [140,141,142]. More recently, TF activation pathway inhibitors have been investigated and prepared for clinical application. These agents specifically target the extrinsic coagulation pathway, paralleling the action of the natural anticoagulant TFPI [1]. In addition, several drugs have been reported to attenuate NET-driven thrombosis in sepsis by inhibiting PADI4 [143] or by reducing NETosis through CXCR1/2 antagonism [144].

The administration of antibodies targeting surface receptors, such as CD14, has effectively reduced complement activation, cytokine release, and hyperinflammatory responses, ultimately improving survival in sepsis models [42]. Similarly, MCC950 has been shown to suppress NLRP3 inflammasome activation, leading to reduced platelet activation and cytokine production in experimental models of sepsis [145,146]. Moreover, Emelin has demonstrated therapeutic potential in attenuating inflammation and thrombosis in sepsis-induced coagulopathy [147]. The antidepressant amitriptyline has also shown anti-inflammatory effects by reducing TNF-α expression and macrophage polarization, thereby inhibiting coagulopathy in mouse models of sepsis [148]. Interestingly, histidine-rich glycoprotein has been found to prevent sepsis-induced immunothrombosis [149].

Several in vivo and in vitro studies have evaluated therapeutic agents for SIC [150], and numerous clinical trials have been conducted. However, most have not demonstrated significant benefits in reducing mortality or disease severity [151,152,153,154]. Clinical use remains limited due to treatment-related complications and minimal impact on coagulation abnormalities [155]. Notably, combination therapies have shown promising results in lowering mortality risk [156,157].

**Table 2:** Commonly used approved drugs for the treatment of sepsis immunothrombosis/thromboinflammation.

Type of Drugs	Target of Drugs	Mechanism	Clinical Relevance	References
Low molecular weight heparins (Bemiparin, Certoparin, Dalteparin, Enoxaparin, Nadroparin, Parnaparin, Reviparin, and Tinzaparin)	Antithrombin III	Anticoagulant and anti-inflammatory	Inhibit coagulation by activating antithrombin III, which binds to and inhibits factor Xa Improve sepsis outcome	[158,159]
Fondaparinux	Thrombin	Anticoagulant	Reduces coagulation in COVID-19 patients	[160,161]
Enoxaparin	Thrombin	Anticoagulant	Reduces coagulation in COVID-19 patients	[160]
Ticlopidine	Platelet P2Y12	Antagonist	Prevents platelet activation (commercially available)	[162]
Clopidogrel	Platelet P2Y12	Antagonist	Prevents platelet activation (commercially available)	[162]
Prasugrel	Platelet P2Y12	Antagonist	Prevents platelet activation (commercially available)	[162]
Ticagrelor	Platelet P2Y12	Antagonist	Prevents platelet activation (commercially available)	[162]
Cangrelor	Platelet P2Y12	Antagonist	Prevents platelet activation (commercially available)	[162]
DNases	NETs	Digest DNA	Prevent platelet activation and coagulation	[26]
Dimetil fumarate	Gasdermin D	Inhibit interaction with caspases	Prevents pyroptosis	[163]
Thrombomodulin	HMGB1	Inhibit HMGB1	Reduces inflammation	[164]
Recombinant human activated protein C	Factor Va and factor VIIIa	Anticoagulant	Reduces mortality in severe sepsis	[150]

**Table 3:** Anti-inflammatory and anticoagulant therapeutic agents under investigation targeting inflammation and coagulation during sepsis-induced immunothrombosis.

Types of Drugs	Target of Drugs	Subject	Outcome	Study Type	References
Eptifibatide	GPIIb/IIIa receptor	Septic shock patients	Reduces endothelial injury, reduces platelet consumption and improves sequential organ failure assessment (SOFA) score	RCT	[140]
MCC950	NLRP3	Sprague Dawley rat treated by cecal ligation and puncture (CLP)	Inhibits NLRP3 activation and reduces platelet activation as well as reduces multi-organ injury	In vivo	[145]
Glycyrrhizin	HMBG1	Mice (C57BL/6) and THP 1 cell	Attenuates caspase-11-dependent immune responses and coagulopathy by inhibiting HMBG1	In vivo and in vitro	[165]
Ticagrelor	NETs	Patient sample and cell line	Prevents platelet activation and coagulation	Ex vivo and in vivo	[166]
Aspirin	Platelets, NF-kB and HMGB1	Sepsis patients	Reduces 30-day mortality	RCT	[141]
Forsythiaside B (FTB) (DNase1, Cl-amidine)	NETs, PAD4	Sprague Dawley rats treated by CLP	Alleviate coagulopathy	In vivo	[97,143]
P2Y12 antagonists (23 chemically synthesized compounds)	Platelets	In vitro	Inhibit platelet activation through P2Y12 antagonist activity	In vitro	[162]
Esaridin E	Integrin αvβ_3_	CLP mice (Male C57BL/6 mice)	Improves endothelial hyperpermeability by inhibiting vWF binding to αvβ3	In vivo	[167]
AntiCD14 antibody	CD14	Baboon (*Papio anubis*)	Reduces activation of complement, proinflammatory cytokines and inflammatory cells	In vivo	[42]
Acetylsalicylic acid (ASA)	Platelets, neutrophils	Mice (C57Bl/6 mice)	Reduces platelet activation, neutrophil recruitment and NET formation	In vivo	[168]
Heparin	Alarmin HMGB1	Mice (*Alb-cre* mice)	Inhibits alarmin HMGB1-LPS interaction and prevents lethal effect of LPS sepsis	In vivo	[158]
Resveratrol-loaded silver nanoparticle	Proinflammatory cytokines	Rat (Sprague Dawley rats)	Reduces proinflammatory cytokines and inhibits activation of NF-kB	In vivo	[169]
Histidine-rich glycoprotein	FXII	Rabbit (Male New Zealand white rabbits), endothelial cell line	Decreases thrombosis associated with the catheter Reduces sepsis-induced shock and DIC	In vitro and In vivo	[149,170,171]
Emelin	Plasminogen activator inhibitor-1 (PAI-1)	Mice (Male Kunming mice)	Alleviates sepsis-induced DIC	In vivo	[147]
Matrine	NLRP3	THP1, J774A.1 cell line and Mice (C57BL/6 mice)	Suppresses of NLRP3 inflammasome activation through regulating protein tyrosine phosphatase non-receptor type 2 (PTPN2)/JNK/SREBP2 signaling	In vitro and In vivo	[85]
Amitriptyline (AMIT)	Inflammatory cytokines (TNF-α)	CLP mice (Male CF-1 outbred mice)	Reduces level of TNF-α and alleviates SIC without bleeding complication	In vivo	[148]
Combination of Probenecid Nanocrystals and Cefotaxime Sodium	Inflammatory cytokines and NETs	Mice (C57BL/6 mice)	Promote sepsis recovery by reducing immunothrombosis formation	In vivo	[157]
Combination of ulinastatin with TIENAM	Inflammatory cytokines	CLP Mice (C57BL/6 mice)	Reduce inflammation and NF-kB pathways suppressed	In vivo	[156]
Parthenolides	Mitochondrial mediated apoptosis	Septic rat (Sprague Dawley rats)	Improves SIC	In vivo	[172]
Nitrofurans, acrylamides, and indole ureas (indole derivatives)	STING	Cell line and mouse model (C57BL/6J mice)	Reduce STING-mediated inflammatory cytokine production	In vitro and in vivo	[163,173]

## 7. Limitations

Although numerous studies have investigated novel therapeutic strategies, no single agent has yet proven to be both safe and effective for managing sepsis-related inflammatory and coagulation disorders. Anticoagulants have long been used in clinical settings, but many are associated with an increased risk of severe bleeding, as documented in multiple studies. The heterogeneity of sepsis pathogenesis in humans further complicates treatment efficacy, often limiting the translational success of agents that show promise in preclinical models. Unlike DNases and other NET-targeted approaches, no clinically approved therapies currently exist that specifically inhibit PADI4. Additionally, some therapeutics exhibit species-specific limitations; for instance, heparin has shown limited efficacy against sepsis caused by Gram-positive bacteria such as *Staphylococcus* spp. The underlying mechanisms of sepsis and its complications remain incompletely understood, highlighting the need for continued basic and translational research. Commonly used animal models, such as CLP in mice, may not fully capture the spectrum of human sepsis, particularly for non-bacterial origins. Variations in species, infection methods, dosages, and experimental conditions may contribute to inconsistent results across studies. These discrepancies often carry over into clinical trials, underscoring the importance of selecting appropriate and representative models for drug development in sepsis and its associated coagulopathies.

## 8. Conclusions

Immunothrombosis represents an interplay between inflammation and coagulation, which aids in the elimination of microorganisms from the body. Hence, fibrin clots trap microorganisms and limit their spread into the systemic circulation. Pathological immunothrombosis, or thromboinflammation, is a hallmark of complicated sepsis, resulting from uncontrolled or hyperinflammatory responses with excessive production of proinflammatory cytokines and chemokines. This dysregulation exacerbates sepsis complications by disrupting the normal process of physiological thrombosis. Further activation of inflammatory cells, including monocytes/macrophages and neutrophils (with or without NETs), platelets, and ECs, leads to the activation of procoagulants and coagulation factors, resulting in excessive thrombus formation, particularly in capillaries. Inflammatory and coagulation processes rely heavily on various intracellular signaling pathways for full activation of the coagulation cascade and formation of both micro- and macrothrombi. Notably, inflammasome pathways such as the NLRP3 inflammasome, along with NF-κB, JAK-STAT, MAPK, and cGAS-STING pathways, play central roles in the intracellular signal transduction responsible for sepsis and SIC. Among molecular regulators, non-coding RNAs, particularly miRNAs, serve as key modulators of inflammatory and coagulation factor expression. Dysregulation of these non-coding RNAs amplifies inflammation and thrombosis, contributing to severe outcomes such as thromboinflammation and DIC. Anti-inflammatory and anticoagulant therapies are frequently employed in the management of SIC, and various therapeutic agents have been identified to target specific stages of cellular activation, intercellular communication, signaling pathways, and the underlying molecular mechanisms driving thrombosis during sepsis. However, many of these agents are associated with limitations, particularly bleeding risks and treatment-related hemorrhage. Therefore, further investigation into safer and more effective therapeutic strategies is warranted to improve the clinical management of sepsis-associated coagulopathic complications.

## Abbreviations

Ag: Antigen; CD14: Cluster of differentiation 14; cGAS-STING: cyclic GMP AMP synthase stimulator of interferon genes; CLP: Cecal ligation and puncture; COVID-19: Coronavirus disease 2019; CXCRs: Chemokine receptors; DAMP: Damage-associated molecular patterns; DIC: Disseminated intravascular coagulation; DNA: Deoxyribonucleic acid; dsDNA: Double-stranded DNA; ECs: Endothelial cells; FVII: Coagulation factor VII; FIX: Coagulation factor IX; FX: Coagulation factor X; FXI: Coagulation factor XI; FXII: Coagulation factor XII; FVIIa: Activated coagulation factor VII; FIXa: Activated coagulation factor IX; FXa: Activated coagulation factor X; FXIa: Activated coagulation factor XI; FXIIa: Activated coagulation factor XII; HAG: heterotypic aggregate; HMGB1: high-mobility group box 1; ICAM: Intercellular adhesion molecule; IL: Interleukin; IL-1β: Interleukin-1β; IRF: Interferon regulatory factor 3; JAK-STAT: Janus kinase-signal transducer and activator of transcription; LPS: Lipopolysaccharide; lncRNAs: long non-coding RNAs; MAPK; mitogen-activated protein kinase; miRNA: microribonucleic acid; MOF: Multi-organ failure; NETs: Neutrophil extracellular traps; NF-kB: Nuclear factor kappa β; NLRP3: NOD like receptor protein 3; P2Y12: P2Y purinoreceptor 12; PAMPs: Pathogen-associated molecular patterns; PDA: platelet-dendritic aggregation; PNA: platelet–neutrophil aggregation; PMA: platelet–monocyte aggregation; PI3K/Akt: Phosphatidylinosital 3 kinase/protein kinase B; PMN: Polymorphonuclear cell; ROS: Reactive oxygen species; SIC: Sepsis induced coagulopathy; SIRS: Systemic inflammatory response syndrome; TF: Tissue factor; TFPI: tissue factor pathway inhibitor; TLRs: Toll-like receptors; TM: Thrombomodulin; TNF: Tumor necrosis factor; VCAM: Vascular cell adhesion molecule; VWF: von Willebrand factor
